# Supplementary material for: Deep-Learning-Based Multivariate Pattern Analysis (dMVPA): A Tutorial and a Toolbox
Source: Front Hum Neurosci. 2021 Mar 2;15:638052. doi: 10.3389/fnhum.2021.638052 (PMC7960649; doi:10.3389/fnhum.2021.638052)
Supplement: Supplementary file 1 [file Data_Sheet_1.PDF]

Example DeLINEATE Analysis Workflow

Below we include an overview on how to get started conducting data analyses with the DeLINEATE toolbox. Because deep learning is continuing to undergo rapid development, with frequent updates to common software packages, detailed guides are apt to go out of date quickly; thus, we have kept the following instructions general enough that they should remain relatively evergreen. For the most detailed and up-to-date information, we recommend users check the DeLINEATE website at http://delineate.it and consult other online installation/configuration guides for the underlying components of the software stack.

Workstation/Environment setup (one-time)

1. If necessary, obtain an appropriate workstation and install operating system
    a. If you wish to perform GPU-accelerated dMVPA, an NVIDIA brand GPU will likely be necessary (although this could change in the future, depending on software updates).
    b. Linux operating systems are recommended for heavy-duty work, but Windows and macOS are also supported.
2. Install basic software dependencies
    a. If performing GPU-accelerated dMVPA: You will need to install the NVIDIA drivers, the CUDA toolkit, and the cuDNN library. These tools are updated frequently; if you are not familiar with deep learning analyses, we recommend searching online for guides. Try to ensure cross-compatibility between the versions of your operating system, NVIDIA driver, CUDA toolkit, cuDNN library, TensorFlow version (assuming you intend to use the TensorFlow backend), and Keras version; because some tools may be updated more often than others, this might require using slightly older versions of some tools than the newest available to maximize compatibility. Once a working configuration is established, we recommend that novices disable automatic software updates and back up a snapshot of their workstation's boot drive; this will reduce the likelihood of accidentally installing an update that is incompatible with the rest of the software stack, but if such an event occurs, a backup will make the previously working configuration easy to restore.
    b. In all cases: Install the Python programming language/environment. We recommend the latest available version of Python 3, if possible, although Python 2 remains supported.
    c. Optional, but recommended: Create a Python "virtual environment" for DeLINEATE, and remember to activate it before installing any Python packages or running any analyses. This will keep any installed packages separate from the system's default Python installation, which will help avoid any conflicts if Python is used for other purposes on that workstation. It will also make it easier to use multiple different versions of the toolbox concurrently without compatibility conflicts (e.g., keeping a legacy version around for replicating old analyses while using the latest version for new experiments), by creating a new virtual environment for each toolbox version. Novices, please seek out online guides for more information on creating virtual environments.
    d. If performing traditional MVPA with PyMVPA: Install PyMVPA. (See the video guides on the DeLINEATE website and/or the instructions on the PyMVPA website for details.)
    e. If performing dMVPA, with or without GPU acceleration: Install a deep learning backend library, followed by the Keras library. At the time of writing, the deep learning library will probably be TensorFlow. (The Theano library is also usable, but is currently deprecated, and thus it is not recommended for novices.) Novices should seek out further information on the DeLINEATE, TensorFlow, and Keras websites.
    f. Download the DeLINEATE toolbox from the http://delineate.it website. This toolbox does not need to be "installed" per se, although you can place it in a directory on your Python installation's search path if desired. Typically, we recommend downloading a fresh copy for each new project and keeping the

toolbox in a directory for that project, alongside your data files (and perhaps your virtual environment folder, if you chose to create one); this ensures that each project always uses the same version of the toolbox even if a toolbox update becomes available during the course of the project, which is good practice for any research workflow.

3. Optional: Configure global settings for your Keras installation. This will involve editing a file named keras.json, located within a .keras directory that should typically be located in the user's home directory. First-time users can likely skip this step, as Keras will generate a default configuration file with reasonable settings for most use cases when it is first run; however, more experienced deep learning practitioners may want to tweak them.
4. Optional, but recommended: Test the toolbox (and the rest of the software/hardware stack) by running one or more of the sample job files included in the DeLINEATE toolbox. For example, to test a Keras-based deep learning analysis:

   python delineate.py sample_jobfiles/sample_jobfile_64x4MLP_customopt.json

   and to test a PyMVPA analysis:

   python delineate.py sample_jobfiles/sample_jobfile_mvpa-smlr.json

   (users may need to adjust these paths relative to their current working directory; for some Python installations, the correct Python command might be *python3* rather than *python*; etc.). If running GPU-accelerated deep learning analyses, you may also wish to open a hardware/process monitor application to ensure that the process is actually using the GPU.

Designing and running a new dMVPA

For this walkthrough, we will assume that the user has already set up their workstation and downloaded/installed/configured/tested DeLINEATE and its dependencies, as described above.

We will also assume that they are using either macOS or Linux (for Windows users, the process would be similar, but we will use Unix-style command-line conventions that would require modest translations for Windows), and that the toolbox is in a folder simply called "delineate" within the user's home directory (i.e., *~/delineate* in Unix parlance). Finally, if the user has chosen to set up a Python virtual environment for DeLINEATE, as recommended above, we assume that the virtual environment has already been activated for all command-line actions described below.

1. Obtain a dataset
   a. If using your own dataset, and you do not wish to write your own loader function, this should be in NumPy .npy or Matlab .mat format; see documentation for details.
   b. Our own example here will use the *sample_fso_dataset_vsl_study.mat* dataset file, included in the DeLINEATE toolbox. This is a portion of an EEG (or event-related potential, ERP) dataset from a visual statistical learning (VSL) experiment in which participants were viewing pictures of faces, scenes, or objects (FSO). Other study details are not too important here; the main question is whether we can classify, based on EEG measurements, which category of stimulus a participant was seeing on a given trial.
   c. Note the dimensions of your dataset. For our sample EEG dataset (see figure below), the file contains a variable named *erp_data* (1912 trials x 18 channels x 273 timepoints) with the data to be classified, as well as a *categories* variable (1912 x 1) with numeric categorical labels for each trial (0, 1, or 2, corresponding to whether a human subject was seeing a face, scene, or object stimulus on the trial in

question). There is also a *subjects* variable in the file, containing subject ID numbers for each trial, but that variable will not be used in this walkthrough.

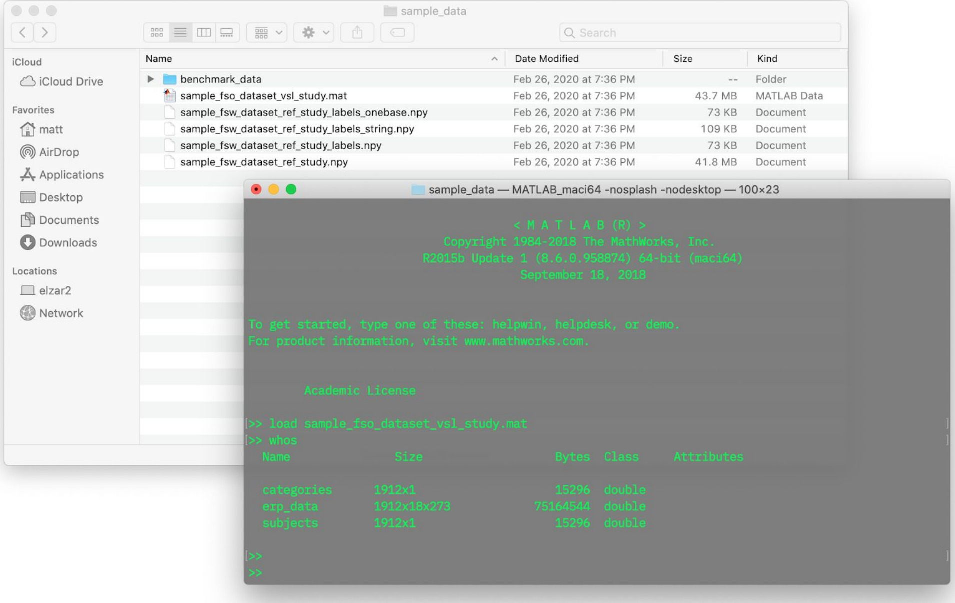

2. Create a JSON-format job file

   a. You can use a dedicated JSON file editor for this task if you want, but any text editor will do. One advantage of a dedicated JSON editor, though, is that it can tell you if there are any syntax errors in your document before you attempt to run it. You can also use the DeLINEATE GUI to configure a job file, but you may want to try editing the text directly before trying the GUI, to get a better understanding of the file format. JSON is a fairly generic file format used to encode many different types of data structures into human-readable text files. If you are not at least passingly familiar with the JSON format, you may want to read up a bit on it online before proceeding further; there are many tutorials available.

   b. In the figure shown here is a very simple neural network for analyzing one of the sample datasets included with the DeLINEATE toolbox. The contents of this file are included at the end of this document as an appendix. In general, you will probably find it easier to start with an existing job file (such as one of the sample job files provided with the toolbox) and modify it, rather than try to create it from scratch, even if you know it will have to be heavily modified.

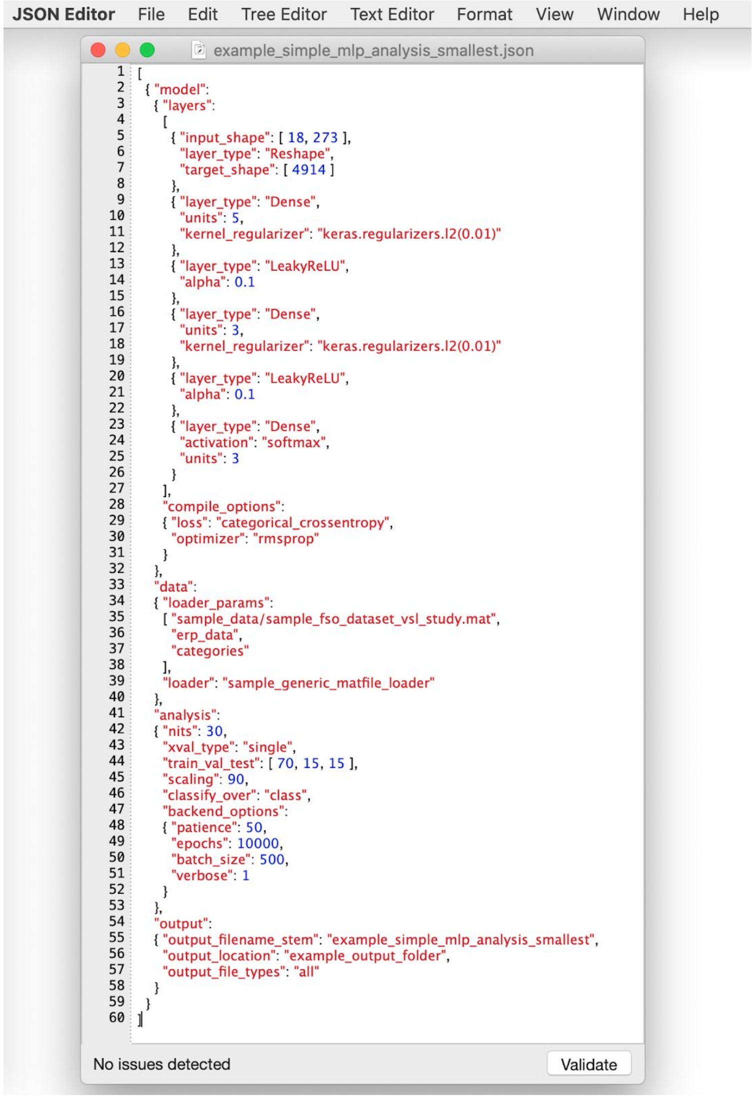

c. We will briefly go through each section of the job file and explain how it works. First, note that the first and last lines are square brackets [ ], which denote a list (or array) in JSON; technically, each job file can contain a list of jobs to run, although all of our examples have only a single job per file. (Also note that the fact that they are on their own lines is only for human readability; the JSON format ignores white space.)

d. Next, note that everything inside those square brackets is again enclosed in curly braces { }, which denote an *object* in JSON, which is roughly equivalent to a *dictionary* in Python; in other words, a set of key/value pairs. (If this vocabulary is all-new to you, you may wish to consult an online guide on JSON and/or Python data types for more description.) Note that values in this dictionary (we will use the Python terminology henceforth) are unordered, i.e., they can appear in any order. A DeLINEATE job always has four keys: *model*, *data*, *analysis*, and *output*, and that is the order they appear in this example, but if you wanted to have the *data* section come before the *model* section in your own files (for example), that would be perfectly fine. (The same holds true for any of the other dictionaries in the job file; the order of the key/value pairs within a dictionary doesn't matter.) Note that each of these four keys is followed by a value; in a DeLINEATE job file, the value is always another dictionary. The values in *those* dictionaries can vary widely depending on what you want to do; essentially, they contain all the various (hyper)parameters required to run an analysis, and there are *many* options to choose from. We will simply go through the choices that are here; see the documentation for a more comprehensive breakdown of options.

e. Let's actually skip the *model* section for a bit and consider the *data* section first, because it is much simpler. There are two keys, *loader* and *loader_params*. For *loader*, we have specified *sample_generic_matfile_loader*, which is the name of the data loader function that will be used to read in our data file. As the name suggests, that function can read in Matlab .mat files, which is what our sample dataset is. It is included with the toolbox; the Python code for it is located in the *sample_loaders.py* file inside the *loaders* directory if you want to see how it works. The value for *loader_params* is a list containing whatever parameters you want passed to the loader function at runtime. What goes in that list depends on which loader function you're using, although in most cases, the first parameter will be a path to your data file. (The path can be absolute, or relative to whatever your current working directory is when you run your analysis.) In this case, we first specified our sample dataset file. For the *sample_generic_matfile_loader* function, the next two parameters should be the name of the Matlab variable in the .mat file that contains the actual data samples (which in this case, as we saw above, is *erp_data*) and then the name of the Matlab variable that contains class labels (in this case, *categories*).

```
"data":
{ "loader_params":
  [ "sample_data/sample_fso_dataset_vsl_study.mat",
    "erp_data",
    "categories"
  ],
  "loader": "sample_generic_matfile_loader"
},
```

f. Now let's skip ahead again to the *output* section, because that is also fairly simple. There are three keys here. For *output_filename_stem*, we have specified *example_simple_mlp_analysis_smallest*; the toolbox will generate a number of output files, but this setting ensures that all output files with this job will be prepended with that string. Next, for *output_location*, we have specified *example_output_folder*. As with any filenames in the file, this can be either an absolute path, or relative to whatever your current working directory is when you run your analysis. (If the folder does not exist already, it will be created for you.) Finally, for *output_file_types*, we have specified *all*, which simply says to generate (almost) all of the output file types that DeLINEATE is capable of creating. This can potentially generate many files; if you want a more limited set of outputs, you can instead specify a list of specific output type codes (e.g., only test accuracies; training and test accuracies; and so on). See the DeLINEATE toolbox documentation for a full rundown of allowable type

```
"output":
{ "output_filename_stem": "example_simple_mlp_analysis_smallest",
  "output_location": "example_output_folder",
  "output_file_types": "all"
}
```

codes. When in doubt, though, *all* is virtually guaranteed to produce the output you want unless you are doing something fairly specialized, and it is easy to remember!

g. Now that you've seen a couple of simpler examples, we'll go back up to the *model* section. If you're running a Keras-based dMVPA, this will likely be the longest section in the job file. If you're running a PyMVPA-based traditional MVPA, the format will be quite a bit different and this section will be significantly shorter. (All of the examples in this document are for dMVPA, but see the *sample_jobfiles* directory in the toolbox and/or the main DeLINEATE documentation for more examples and details on how to configure a PyMVPA analysis.)

```
{ "model":
  { "layers":
    [
      { "input_shape": [ 18, 273 ],
        "layer_type": "Reshape",
        "target_shape": [ 4914 ]
      },
      { "layer_type": "Dense",
        "units": 5,
        "kernel_regularizer": "keras.regularizers.l2(0.01)"
      },
      { "layer_type": "LeakyReLU",
        "alpha": 0.1
      },
      { "layer_type": "Dense",
        "units": 3,
        "kernel_regularizer": "keras.regularizers.l2(0.01)"
      },
      { "layer_type": "LeakyReLU",
        "alpha": 0.1
      },
      { "layer_type": "Dense",
        "activation": "softmax",
        "units": 3
      }
    ],
    "compile_options":
    { "loss": "categorical_crossentropy",
      "optimizer": "rmsprop"
    }
  },
```

First, let's cover the *compile_options* part, because that's shorter. The value for *compile_options* should be another dictionary of values covering options that are relevant to your entire neural network (not just one layer) when it is "compiled" by Keras before training. The format of this can get a bit hairy; see our documentation for details. In this particular configuration, the only compile options listed are *loss*, which is the type of loss function to use (i.e., the prediction-error value you are trying to minimize while training the model; not all loss functions are good fits for all types of analyses, so for most standard classification analyses you may just want to stick with *categorical_crossentropy*), and *optimizer*, which is the algorithm that determines exactly how to adjust the network weights during training (there are various choices that can be reasonable; two we sometimes use include *rmsprop*, as is used here, and *Adam*). Note that depending on which optimizer you choose, you may want to specify some additional optimizer options (see further below for one example), but we aren't doing that here.

Now, to *layers*, which takes the form of a list of dictionaries. Each dictionary represents one layer of a sequential neural network. As such, the order of the dictionaries in the list matters; input layer at the top, then middle layers in order, and the output layer at the bottom. This network is a very small and relatively simple fully-connected network, a.k.a. a network of Dense layers, a.k.a. a multi-layer perceptron. If you are not familiar with the neural network layer types in Keras (or neural network architectures in general), now would be an excellent time to take an intermission from this document and consult a few online tutorials in those topics. [pause] Welcome back! The first layer in this particular network is a basic utility layer that simply reshapes the data, because each trial of our input data is an 18x273 matrix (as noted in the *input_shape* key, which must be specified for the first layer in each network; after the first layer, Keras can calculate layer input/output sizes automatically), but we want to reshape that into a vector because our network only has Dense layers in it, and Dense layers (unlike, for example, convolutional layers) only take vectorized input. We specify that this is a *Reshape* layer with the *layer_type* key, and specify that the output should be a 4914-length vector (18 times 273) by putting a list with the single integer *4914* after the *target_shape* key.

Next we are ready for our first "real" layer, a Dense layer. (In other words, a layer of simple fully-connected perceptron units.) This layer will have *units* = 5 (i.e., 5 artificial neurons) and we will also use *kernel_regularizer* to add a small L2 regularization penalty to the synaptic weights of the unit, which can sometimes help avoid overfitting. (The syntax details for specifying regularizers are somewhat

hairy; please see the documentation for further details.) Each “main” layer of the network must also have an activation function that determines how the inputs are summed and thresholded to produce an output. This activation function can be specified either as a hyperparameter of the Dense layer or as a standalone layer immediately after it; we have chosen the latter here. For this activation function/layer we chose a leaky rectified linear unit or *LeakyReLU*; there are several options for activation layers in Keras, but *LeakyReLU* is often a reasonable default choice. We have set the *alpha* hyperparameter of that layer, which determines the function’s slope for input values < 0, to 0.1.

Following the first 5-unit Dense layer, we have a very similarly-configured Dense layer with another *LeakyReLU* activation function; however, this second Dense layer only has 3 units in it. After that, our last layer is one more Dense layer, again with 3 units. Because it is the last layer, Keras implicitly considers it the output layer. Note that if you are doing a typical multiclass classification analysis, the number of units in the output layer should match the number of classes. Also, because in output layers we often want to normalize the output scores to sum to 1 so they can be interpreted as probability scores, we are using a *softmax* activation function (note that in this case, the activation function is specified as part of the Dense layer, which is more succinct when the activation function does not need any additional hyperparameters specified).

h. Lastly, we’ll review the *analysis* section of the job file. This section contains a variety of global configuration options for the entire analysis (i.e., ones not specific to a particular neural network model or dataset). Note that some of these options are specific to Keras or PyMVPA, whereas others are specified similarly for both backends. Here, we have specified a *nits* (number of iterations) value of 30, which means that we will run 30 iterations of cross-validation, selecting a different random training/validation/test set each time. The *xval_type* (short for cross-validation type) is *single*, which in our type coding system (see toolbox documentation for additional types) means to treat the dataset as a single pool (e.g., not run separate analyses for each subject) and choose the training/validation/test sets randomly from that pool. The *train_val_test* is a three-item list of 70, 15, and 15, indicating that on each fold of the cross-validation, 70% of the data will be used for training, 15% for validation (used to stop training when performance on the validation set reaches asymptote), and 15% for testing. The *scaling* is set to 90, which means that all of the data on each cross-validation iteration will be scaled by dividing it by the value at the 90th percentile of the absolute value of all data points in the training data. (There are several different normalization/scaling methods available; normally a *scaling_method* parameter would be present as well, but when only *scaling* is specified, the scaling method defaults to percentile-based scaling.) The *classify_over* setting is set to *class*, which represents the name of the “sample attribute” variable in the dataset to be used as category/class labels for classification. (Astute readers may remember that the label variable was originally named *categories* in the Matlab data file, but our loader function automatically renames the label variable to *class* during the import process.) Finally, there is the *backend_options* parameter, which specifies a dictionary of backend-specific options (in this case, Keras-specific). These values include *patience*, set to 50, which means that training of the neural network will be stopped when performance on the held-out validation set has not improved for 50 training epochs; *epochs*, set to 10000, which is effectively an arbitrarily high number, as we expect in virtually any case that the *patience* option will cause early stopping before the *epochs* limit is reached; *batch_size*, set to 500, which controls the size of mini-batches of training examples (this setting can have effects on memory usage, speed, and accuracy, but a full discussion is beyond the scope of this document; see other online tutorials for details); and *verbose*, set to 1, which controls how much text output is displayed to the terminal during training (see Keras and DeLINEATE documentation for details).

```
"analysis":
{ "nits": 30,
  "xval_type": "single",
  "train_val_test": [ 70, 15, 15 ],
  "scaling": 90,
  "classify_over": "class",
  "backend_options":
  { "patience": 50,
    "epochs": 10000,
    "batch_size": 500,
    "verbose": 1
  }
},
```

    i. Once you have chosen all of your settings, it is probably a good idea to double-check the syntax of your JSON-format job file. Although JSON is a very readable format for human eyes, it is also very easy to make trivial mistakes that humans pass over but computers balk at. (In our experience, omitting a comma or including one where it shouldn't be is probably the most common source of JSON syntax errors.) Here it is helpful to have either a JSON-dedicated application or at least a text editor that is aware enough of the JSON format to highlight syntax errors.

3. Run the analysis!
    a. Open a terminal window.
    b. If you are using a Python virtual environment, as we recommend, remember to activate that virtual environment.
    c. Change into the DeLINEATE toolbox directory:

        cd ~/delineate

        As noted earlier, we will assume Unix command-line conventions and that the toolbox has been installed into a folder named "delineate" in the user's home directory. DeLINEATE can be run from any directory on job files located anywhere, but we will also assume, for the sake of simplicity and making the terminal commands we print take up less space, that you have saved your JSON job file inside that "delineate" folder. However, in actual practice, it is probably a better idea to store your job files and results somewhere else, and leave the toolbox directory pristine as it was originally downloaded.
    d. Assuming your JSON job file is named *example_simple_mlp_analysis_smallest.json* as in the screenshot above, you can run the analysis with the following command:

        python delineate.py example_simple_mlp_analysis_smallest.json

        (Note/recall that in some installations, your Python interpreter may have a slightly different name, like *python3* instead of *python*.)
    e. Troubleshoot! When crafting a new analysis job from scratch, it is fairly likely that errors will occur. Assuming you have already checked JSON syntax and your file technically contains valid JSON, typos in the names or values of various hyperparameters are likely sources of problems. If you are using your own data or a different network structure, mismatches between the actual shape of your dataset and the purported *input_shape* of your first network layer can also be an issue (e.g., if you got the order of the dimensions backwards). Newer users may also be more likely to specify impossible sequences of layer types or to have forgotten to specify required hyperparameters. In general, we have tried to make error messages as informative as possible, although sometimes it is difficult for our toolbox to interpret an error that occurs within Keras due to a mis-configured network. In any case, if you are unable to immediately discern the source of the error, keep the faith and double-check your JSON file line by line. If all else fails, consider starting with a job file that is known to work correctly and gradually change its settings, testing after each change, until you have successfully transformed it into a working version of your intended analysis.

f. When your analysis job is successfully running, you should see output that looks something like this:

delineate — Python delineate.py example_simple_mlp_analysis_smallest.json — 140×42

```
delineate.py: running json file number 1 of 1
DeLINEATE: Iteration 1
2021-01-28 21:12:50.729471: I tensorflow/compiler/jit/xla_cpu_device.cc:41] Not creating XLA devices, tf_xla_enable_xla_devices not set
2021-01-28 21:12:50.729699: I tensorflow/core/platform/cpu_feature_guard.cc:142] This TensorFlow binary is optimized with oneAPI Deep Neural
 Network Library (oneDNN) to use the following CPU instructions in performance-critical operations:  AVX2 FMA
To enable them in other operations, rebuild TensorFlow with the appropriate compiler flags.
Model: "sequential"
_________________________________________________________________
Layer (type)                 Output Shape              Param #
=================================================================
reshape (Reshape)            (None, 4914)              0
_________________________________________________________________
dense (Dense)                (None, 5)                 24575
_________________________________________________________________
leaky_re_lu (LeakyReLU)      (None, 5)                 0
_________________________________________________________________
dense_1 (Dense)              (None, 3)                 18
_________________________________________________________________
leaky_re_lu_1 (LeakyReLU)    (None, 3)                 0
_________________________________________________________________
dense_2 (Dense)              (None, 3)                 12
=================================================================
Total params: 24,605
Trainable params: 24,605
Non-trainable params: 0
_________________________________________________________________
2021-01-28 21:12:50.819315: I tensorflow/compiler/mlir/mlir_graph_optimization_pass.cc:116] None of the MLIR optimization passes are enabled
 (registered 2)
Epoch 1/10000
3/3 [==============================] - 1s 210ms/step - loss: 1.5876 - acc: 0.3575 - val_loss: 1.2360 - val_acc: 0.3978
Epoch 2/10000
3/3 [==============================] - 0s 10ms/step - loss: 1.2095 - acc: 0.4404 - val_loss: 1.3233 - val_acc: 0.3477
Epoch 3/10000
3/3 [==============================] - 0s 10ms/step - loss: 1.2748 - acc: 0.4499 - val_loss: 1.2810 - val_acc: 0.4229
Epoch 4/10000
3/3 [==============================] - 0s 11ms/step - loss: 1.2499 - acc: 0.4640 - val_loss: 1.2031 - val_acc: 0.4337
Epoch 5/10000
3/3 [==============================] - 0s 11ms/step - loss: 1.1374 - acc: 0.5254 - val_loss: 1.1906 - val_acc: 0.4588
Epoch 6/10000
3/3 [==============================] - 0s 10ms/step - loss: 1.0993 - acc: 0.5570 - val_loss: 1.2544 - val_acc: 0.3978
Epoch 7/10000
3/3 [==============================] - 0s 11ms/step - loss: 1.1937 - acc: 0.4990 - val_loss: 1.1926 - val_acc: 0.4409
```

The first two lines shown here state that this is the first of one job file passed in (it is also possible to run multiple job files in one command, although we won't demonstrate that now), and that we are on the first cross-validation iteration (although Python typically uses zero-based counting, this iteration counter displays in more human-readable one-based counting). After that are some informational messages from the TensorFlow backend that can be ignored.

Next we see a text diagram of our neural network (Reshape layer, Dense layer, etc.), which should match what we intended to create in the *model* section of our job file. This ends with a summary of how many (trainable) parameters are in the model – in this case, 24,605 – which gives a rough idea of the complexity of the architecture.

After that, we may see some more ignorable informational text from TensorFlow, followed by results from each epoch of training the network. This includes the epoch number, a progress bar for that training epoch, a timer of how long completed epochs took to train, and both loss and accuracy values for the training data and the validation data. Depending on how quickly the training goes in each epoch, they may scroll rather quickly. (In this example, with a very simple multi-layer perceptron network, each epoch takes less than one second to execute, even running on a Mac computer without GPU acceleration.) When performance on the validation set has stopped improving, these per-epoch results will stop, the trained model will be run on the test dataset (which does not produce any visible terminal output, but does write out results to data files), and the process will then repeat with the next iteration of cross-validation.

g. As the training proceeds, you may want to keep an eye on the per-epoch displays to assess how well your model seems to be working:

```
delineate — -zsh — 140×42
Epoch 15/10000
3/3 [==============================] - 0s 16ms/step - loss: 0.9540 - acc: 0.6291 - val_loss: 1.1791 - val_acc: 0.4337
Epoch 16/10000
3/3 [==============================] - 0s 15ms/step - loss: 0.9502 - acc: 0.6234 - val_loss: 1.1831 - val_acc: 0.4910
Epoch 17/10000
3/3 [==============================] - 0s 15ms/step - loss: 0.9140 - acc: 0.6646 - val_loss: 1.1657 - val_acc: 0.5090
Epoch 18/10000
3/3 [==============================] - 0s 12ms/step - loss: 0.9381 - acc: 0.6552 - val_loss: 1.2430 - val_acc: 0.4695
Epoch 19/10000
3/3 [==============================] - 0s 12ms/step - loss: 0.9361 - acc: 0.6542 - val_loss: 1.1841 - val_acc: 0.4803
Epoch 20/10000
3/3 [==============================] - 0s 12ms/step - loss: 0.9066 - acc: 0.6539 - val_loss: 1.1698 - val_acc: 0.5125
Epoch 21/10000
3/3 [==============================] - 0s 12ms/step - loss: 0.8784 - acc: 0.7070 - val_loss: 1.1868 - val_acc: 0.4839
Epoch 22/10000
3/3 [==============================] - 0s 13ms/step - loss: 0.9376 - acc: 0.6337 - val_loss: 1.1573 - val_acc: 0.5090
Epoch 23/10000
3/3 [==============================] - 0s 12ms/step - loss: 0.8881 - acc: 0.6761 - val_loss: 1.1980 - val_acc: 0.5090
Epoch 24/10000
3/3 [==============================] - 0s 12ms/step - loss: 0.8895 - acc: 0.6683 - val_loss: 1.1866 - val_acc: 0.4659
Epoch 25/10000
3/3 [==============================] - 0s 13ms/step - loss: 0.8981 - acc: 0.6624 - val_loss: 1.1522 - val_acc: 0.5125
Epoch 26/10000
3/3 [==============================] - 0s 12ms/step - loss: 0.8591 - acc: 0.6990 - val_loss: 1.1685 - val_acc: 0.5197
Epoch 27/10000
3/3 [==============================] - 0s 12ms/step - loss: 0.8671 - acc: 0.6872 - val_loss: 1.1524 - val_acc: 0.5090
Epoch 28/10000
3/3 [==============================] - 0s 12ms/step - loss: 0.8541 - acc: 0.6838 - val_loss: 1.1699 - val_acc: 0.5018
Epoch 29/10000
3/3 [==============================] - 0s 12ms/step - loss: 0.8271 - acc: 0.7085 - val_loss: 1.1691 - val_acc: 0.5341
Epoch 30/10000
3/3 [==============================] - 0s 12ms/step - loss: 0.8433 - acc: 0.7108 - val_loss: 1.2762 - val_acc: 0.4803
Epoch 31/10000
3/3 [==============================] - 0s 12ms/step - loss: 0.8982 - acc: 0.6702 - val_loss: 1.1555 - val_acc: 0.5233
Epoch 32/10000
3/3 [==============================] - 0s 11ms/step - loss: 0.8324 - acc: 0.7109 - val_loss: 1.2215 - val_acc: 0.5054
Epoch 33/10000
3/3 [==============================] - 0s 12ms/step - loss: 0.8254 - acc: 0.7195 - val_loss: 1.1576 - val_acc: 0.5054
Epoch 34/10000
3/3 [==============================] - 0s 12ms/step - loss: 0.8103 - acc: 0.7227 - val_loss: 1.1762 - val_acc: 0.4946
Epoch 35/10000
3/3 [==============================] - 0s 12ms/step - loss: 0.8472 - acc: 0.6887 - val_loss: 1.1620 - val_acc: 0.5305
```

It is hard to generalize based on training performance, but there are a few things that can be said that should be true in most cases. First of all, although you should really only interpret results from your fully held-out test dataset to avoid double-dipping issues, the validation accuracy is usually a fairly good approximation of what you can expect from your test accuracy (unless your dataset is rather small, and random sampling fluctuations result in much higher quality data going into the validation set than the test set, or vice versa). Thus, if validation accuracy is stuck at chance even after a large number of training epochs, you may wish to cancel the analysis prematurely (using Control-C should work to terminate the script, as in most command-line applications) and re-evaluate your neural network architecture.

In this case, chance is 0.333 (repeating, of course), so both training and validation accuracy are comfortably above chance, though also far from perfect. Although, again, overgeneralization is unwise, there are a few things that you *might* be able to infer from the training accuracies, the validation accuracies, and the relationship between them:

• If training accuracy is at chance even after a number of epochs (fairly uncommon), your data may simply be bad, with little decodable signal. Or, your network architecture could just be way off, perhaps with entirely the wrong number/type/size of layers.

• If training accuracy and validation accuracy are both above chance, but very close to the same sub-perfect value (for example, both stuck right around 60%), this could be a sign that your network architecture is too simple and you are underfitting. Most well-trained neural networks overfit the training

data at least a small amount; if yours doesn't, it may mean that the network doesn't have enough trainable parameters to account for the complexity in the data. Consider adding more layers, or perhaps increasing the number of units in the layers you have. Other architecture tweaks might be appropriate as well. Alternately, though, this scenario could also just mean that 40% of your trials are pure noise, and the network is doing perfectly on the 60% that actually contain good signal.

• If your training accuracy is perfect/near-perfect but your validation accuracy is nowhere near that good, you are probably overfitting. This is not *necessarily* a bad thing; sometimes validation/test accuracy can still be very good even when the network is clearly overfitting on the training data. However, it could be a sign that you would do better by simplifying (or otherwise reconfiguring) your network so that it tends to learn more generalizable patterns in the data rather than simply memorizing the training data. Of course, if your training accuracy is perfect but your validation accuracy is at *chance*, then that is an even more dire sign of overfitting – your network is merely memorizing the training data and learning no generalizable patterns at all. (In this case you might still do better if you simplify/reconfigure the network – or it could be a sign that your data do not have any generalizable patterns to learn in the first place.)

• If your training accuracy and validation accuracy are both above chance, and your training accuracy is not terribly far above your validation accuracy (as is the case in the figure above), you are probably in reasonably good shape in terms of overfitting versus underfitting. As noted above, a well-trained network will often overfit at least a little, because it is nearly impossible to capture exactly the right network settings to learn *only* the generalizable patterns in the data but *absolutely nothing more*. However, if you see this situation, it doesn't necessarily mean your network is the best it could possibly be; there could still be tweaks to the network architecture/options that could improve performance on both training and validation. It just means you probably do not have serious issues with either overfitting or underfitting.

• If your training accuracy and validation accuracy are both perfect, your work here is done, and you should probably be the one writing the tutorial. Or else you have chosen a dataset that is too easily classified to be interesting.

Once more, we highly recommend that you do not take these rules of thumb as absolute truths; there are certainly situations that could break the assumptions underlying all of them. As always, it will help tremendously to be familiar with your data and, if possible, have some reasonable prior expectations about how well it might be possible to classify that dataset, either based on previous studies or on the performance of simpler classifiers (e.g., traditional MVPA), or both. The heuristics can help, but they can't take the place of thorough knowledge and careful logic.

h. When all iterations have ended, you can check out your results. There are many types of output that can be produced with the DeLINEATE toolbox, but for a rough summary of how a classifier is working, there is a simple accuracy summarizer script you can run with:

python utilities/dt_accs_summarizer.py example_output_folder/example_simple_mlp_analysis_smallest_accs.tsv

(This is assuming you still have the main toolbox directory as your current working directory.) This will produce output that looks like this:

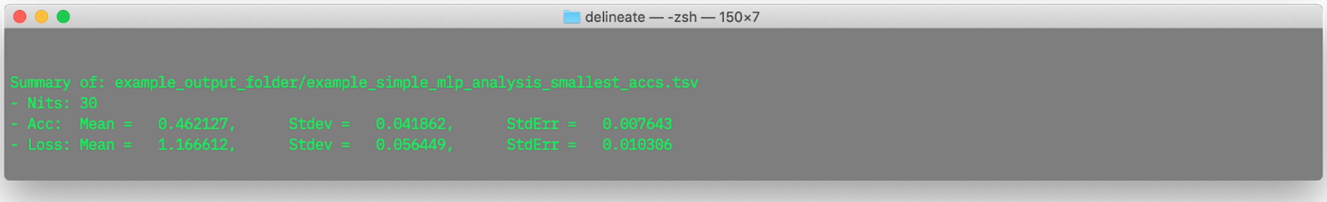

It simply summarizes the number of iterations and the mean, standard deviation, and standard error of

the accuracy and loss values for the test dataset. Here, we can see that the mean accuracy was 46.2% (again, against chance accuracy of 33.3%), with a standard deviation across cross-validation iterations of about 4.2%.

i. You may also want to check out your other output, which looks something like this:

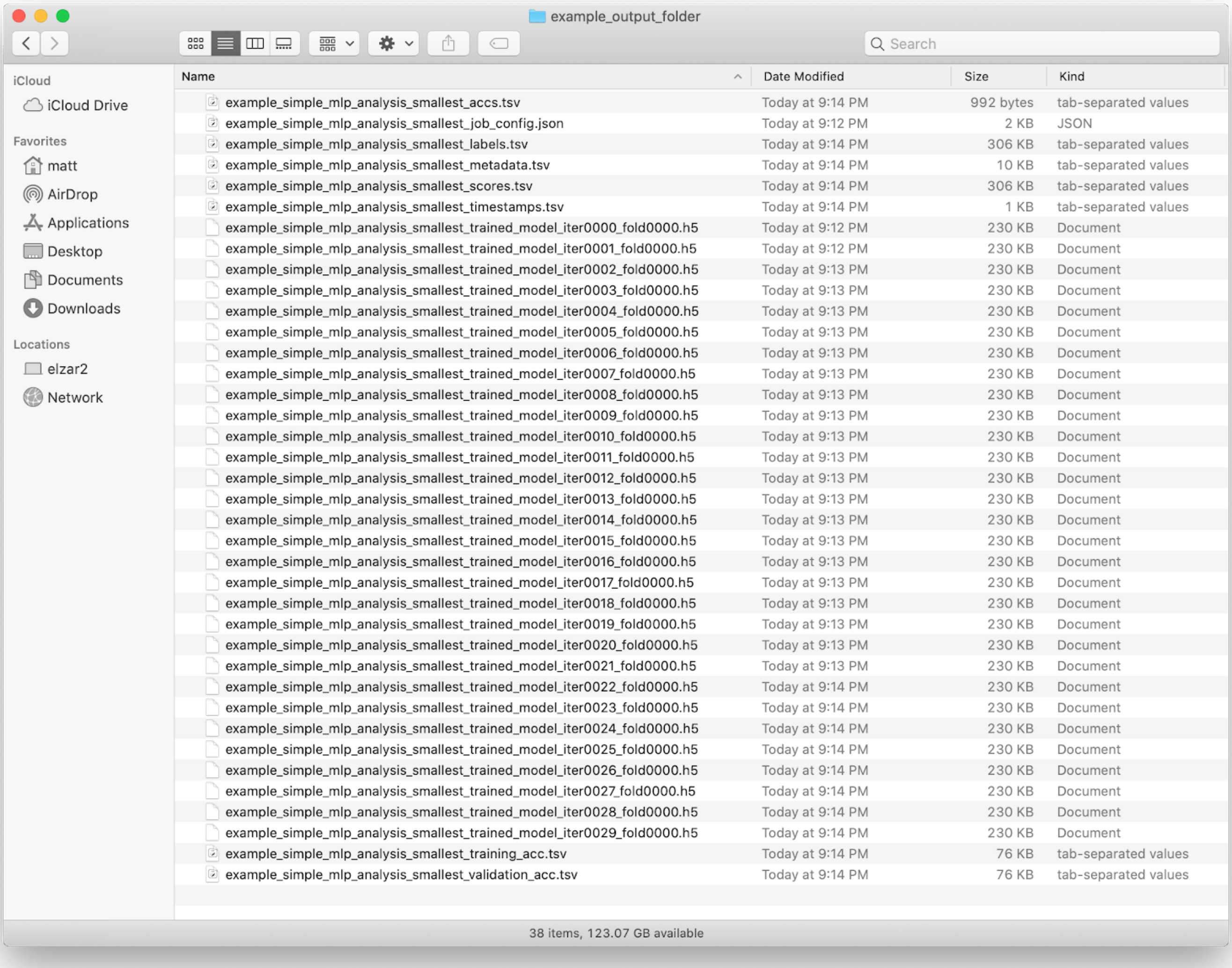

These outputs include tab-separated value (TSV) files for the test set accuracies/losses of each iteration (simply appended with _*accs* because test accuracies are usually the main accuracies of interest), category labels for the trials in the test set for each iteration (_*labels*), miscellaneous metadata about the hardware and software versions used to conduct the analysis (_*metadata*), per-category output classification scores (unthresholded) for the trials in the test set for each iteration (_*scores*), timestamps of when each iteration completed (_*timestamps*), and accuracies/losses for training and validation for each iteration (_*training_acc* and _*validation_acc*, respectively). There is also a copy of the JSON job file used to run the analysis (useful for record-keeping in case the original is accidentally renamed or altered) and a copy of the final trained network for each iteration of cross-validation (all of the *.h5* files), so that the trained network can potentially be reloaded and applied to new data in the future.

4. Experiment with variations on that analysis

   a. Now, let's try a few modifications and tweaks on that very simple/small neural network, to see if we can increase accuracy above the 46.2% baseline we established above. (Of course, this is just a sample dataset, so there is no concern about p-hacking. Whether p-hacking is a concern for a real analysis will

depend somewhat on the nature of your data, the intent of your study, and the standards of your research field.) First, we'll try increasing the complexity of the neural network – not changing the number or type of layers, but simply adding units to the existing layers:

example_simple_mlp_analysis_midsize.json

```
[
 { "model":
   { "layers":
     [
      { "input_shape": [ 18, 273 ],
        "layer_type": "Reshape",
        "target_shape": [ 4914 ]
      },
      { "layer_type": "Dense",
        "units": 32,
        "kernel_regularizer": "keras.regularizers.l2(0.01)"
      },
      { "layer_type": "LeakyReLU",
        "alpha": 0.1
      },
      { "layer_type": "Dense",
        "units": 12,
        "kernel_regularizer": "keras.regularizers.l2(0.01)"
      },
      { "layer_type": "LeakyReLU",
        "alpha": 0.1
      },
      { "layer_type": "Dense",
        "activation": "softmax",
        "units": 3
      }
```

Here, we are only showing the parts that have changed; we have increased the number of units in the first Dense layer from 5 to 32, and the second Dense layer from 3 to 12. (In the bottom part of the JSON file, not shown here, we also changed the *output_filename_stem* to make sure we write out a fresh set of appropriately-named output files.) When we run this job file, we can see the new network information:

delineate — Python delineate.py example_simple_mlp_analysis_midsize.json — 140×42

```
delineate.py: running json file number 1 of 1
DeLINEATE: Iteration 1
2021-01-28 21:15:54.846270: I tensorflow/compiler/jit/xla_cpu_device.cc:41] Not creating XLA devices, tf_xla_enable_xla_devices not set
2021-01-28 21:15:54.846505: I tensorflow/core/platform/cpu_feature_guard.cc:142] This TensorFlow binary is optimized with oneAPI Deep Neural
 Network Library (oneDNN) to use the following CPU instructions in performance-critical operations:  AVX2 FMA
To enable them in other operations, rebuild TensorFlow with the appropriate compiler flags.
Model: "sequential"
_________________________________________________________________
Layer (type)                 Output Shape              Param #
=================================================================
reshape (Reshape)            (None, 4914)              0
_________________________________________________________________
dense (Dense)                (None, 32)                157280
_________________________________________________________________
leaky_re_lu (LeakyReLU)      (None, 32)                0
_________________________________________________________________
dense_1 (Dense)              (None, 12)                396
_________________________________________________________________
leaky_re_lu_1 (LeakyReLU)    (None, 12)                0
_________________________________________________________________
dense_2 (Dense)              (None, 3)                 39
=================================================================
Total params: 157,715
Trainable params: 157,715
Non-trainable params: 0
_________________________________________________________________
2021-01-28 21:15:54.934801: I tensorflow/compiler/mlir/mlir_graph_optimization_pass.cc:116] None of the MLIR optimization passes are enabled
 (registered 2)
Epoch 1/10000
3/3 [==============================] - 1s 227ms/step - loss: 2.6343 - acc: 0.3378 - val_loss: 2.2701 - val_acc: 0.3477
Epoch 2/10000
3/3 [==============================] - 0s 14ms/step - loss: 1.9694 - acc: 0.4300 - val_loss: 1.8322 - val_acc: 0.3799
Epoch 3/10000
```

The overall structure is the same as before, except for the number of units in each layer and the number of parameters, which has grown from the previous value of 24,605 to a new value of 157,715.

When that is finished running, we can run the accuracy summarizer we ran before, and find out that our new network produces an average test accuracy of 48.8%, an increase of about 1.6%. This is not enormous, but if we open up the two test accuracy files in a statistics software package and t-test them against each other, we find that the difference is statistically significant ($p<.01$).

b. Now we have a new baseline of 48.8% accuracy to beat. Perhaps we can try increasing the size of the network again?

example_simple_mlp_analysis_largest.json

```
[
 { "model":
  { "layers":
    [
     { "input_shape": [ 18, 273 ],
       "layer_type": "Reshape",
       "target_shape": [ 4914 ]
     },
     { "layer_type": "Dense",
       "units": 128,
       "kernel_regularizer": "keras.regularizers.l2(0.01)"
     },
     { "layer_type": "LeakyReLU",
       "alpha": 0.1
     },
     { "layer_type": "Dense",
       "units": 48,
       "kernel_regularizer": "keras.regularizers.l2(0.01)"
     },
     { "layer_type": "LeakyReLU",
       "alpha": 0.1
     },
     { "layer_type": "Dense",
       "activation": "softmax",
       "units": 3
     }
    ],
```

Now we have 128 units in the first Dense layer and 48 in the second. This increases the number of parameters even more:

delineate — Python delineate.py example_simple_mlp_analysis_largest.json — 140×42

```
delineate.py: running json file number 1 of 1
DeLINEATE: Iteration 1
2021-01-28 21:19:48.163908: I tensorflow/compiler/jit/xla_cpu_device.cc:41] Not creating XLA devices, tf_xla_enable_xla_devices not set
2021-01-28 21:19:48.164107: I tensorflow/core/platform/cpu_feature_guard.cc:142] This TensorFlow binary is optimized with oneAPI Deep Neural
 Network Library (oneDNN) to use the following CPU instructions in performance-critical operations:  AVX2 FMA
To enable them in other operations, rebuild TensorFlow with the appropriate compiler flags.
Model: "sequential"
_________________________________________________________________
Layer (type)                 Output Shape              Param #
=================================================================
reshape (Reshape)            (None, 4914)              0
_________________________________________________________________
dense (Dense)                (None, 128)               629120
_________________________________________________________________
leaky_re_lu (LeakyReLU)      (None, 128)               0
_________________________________________________________________
dense_1 (Dense)              (None, 48)                6192
_________________________________________________________________
leaky_re_lu_1 (LeakyReLU)    (None, 48)                0
_________________________________________________________________
dense_2 (Dense)              (None, 3)                 147
=================================================================
Total params: 635,459
Trainable params: 635,459
Non-trainable params: 0
_________________________________________________________________
2021-01-28 21:19:48.258067: I tensorflow/compiler/mlir/mlir_graph_optimization_pass.cc:116] None of the MLIR optimization passes are enabled
 (registered 2)
Epoch 1/10000
3/3 [==============================] - 1s 248ms/step - loss: 6.1917 - acc: 0.3533 - val_loss: 6.4890 - val_acc: 0.3226
Epoch 2/10000
3/3 [==============================] - 0s 21ms/step - loss: 5.6193 - acc: 0.4081 - val_loss: 4.4664 - val_acc: 0.4122
Epoch 3/10000
3/3 [==============================] - 0s 22ms/step - loss: 4.2316 - acc: 0.4836 - val_loss: 4.0086 - val_acc: 0.5054
Epoch 4/10000
3/3 [==============================] - 0s 24ms/step - loss: 3.8584 - acc: 0.5585 - val_loss: 3.8087 - val_acc: 0.4731
```

Now we have over 600,000 parameters, about four times as many as in the previous analysis. When we finish running the analysis and check the mean test accuracy, it's 50.2%, another small increase of about 1.4%, but again statistically significant (p=.036).

c. That larger network did perform a bit better, but it took a lot of resources to do so, and more time… the midsize network took around 3.5 minutes to run 30 cross-validation iterations on an early 2020 MacBook Air (without GPU acceleration), whereas the larger network took around 13 minutes. So perhaps let's see if we can work smarter, not harder, and achieve similar results by tweaking other aspects of the midsize network. For example, let's try adding dropout between layers of our network:

example_simple_mlp_analysis_midsize_dropout.json

```
[
 { "model":
   { "layers":
     [
       { "input_shape": [ 18, 273 ],
         "layer_type": "Reshape",
         "target_shape": [ 4914 ]
       },
       { "layer_type": "Dense",
         "units": 32,
         "kernel_regularizer": "keras.regularizers.l2(0.01)"
       },
       { "layer_type": "LeakyReLU",
         "alpha": 0.1
       },
       { "layer_type": "Dropout",
         "rate": 0.3
       },
       { "layer_type": "Dense",
         "units": 12,
         "kernel_regularizer": "keras.regularizers.l2(0.01)"
       },
       { "layer_type": "LeakyReLU",
         "alpha": 0.1
       },
       { "layer_type": "Dropout",
         "rate": 0.3
       },
       { "layer_type": "Dense",
         "activation": "softmax",
         "units": 3
       }
```

Dropout essentially randomly drops a certain percentage of units during training, which injects some unpredictability into the training process and acts like another form of regularization. This, in turn, can sometimes decrease overfitting and thus increase generalizability. We add dropout by inserting new Dropout layers between the output of one Dense layer (i.e., the LeakyReLU layer) and the next Dense layer. Here, we use a dropout rate of 0.3 or 30%, so almost 1/3 of units will be randomly ignored during each training epoch.

When we run this job file, we find an average accuracy of 50.2% – exactly the same as our largest network without dropout. (Coincidentally, in our test runs, they really did come out exactly the same down to several more decimal places, but if you replicate those tests, you will probably get slightly different results, due to the fact that various elements of the training process rely on some amount of randomization and will thus come out slightly differently from one run to the next.) However, the mid-size network with dropout ran much faster, in about the same 3.5 minute span as the mid-size network without dropout.

d. Now let's see if we can tweak something else to improve yet further on the midsize network with dropout. For instance, let's try adding batch normalization, which is a somewhat more complicated concept than dropout. Thus, for the details, we refer you to various online tutorials, but suffice to say it, too, can act like a form of regularization to improve network performance, and it can also significantly speed up run times in some cases. Here is a modification of the last batch file, now with batch normalization added:

example_simple_mlp_analysis_midsize_dropout_batchnorm.json

```
[
 { "model":
  { "layers":
   [
    { "input_shape": [ 18, 273 ],
     "layer_type": "Reshape",
     "target_shape": [ 4914 ]
    },
    { "layer_type": "Dense",
     "units": 32,
     "kernel_regularizer": "keras.regularizers.l2(0.01)"
    },
    { "layer_type": "LeakyReLU",
     "alpha": 0.1
    },
    { "layer_type": "Dropout",
     "rate": 0.3
    },
    { "layer_type": "BatchNormalization"
    },
    { "layer_type": "Dense",
     "units": 12,
     "kernel_regularizer": "keras.regularizers.l2(0.01)"
    },
    { "layer_type": "LeakyReLU",
     "alpha": 0.1
    },
    { "layer_type": "Dropout",
     "rate": 0.3
    },
    { "layer_type": "BatchNormalization"
    },
    { "layer_type": "Dense",
     "activation": "softmax",
     "units": 3
    }
   ],
```

As you can see, batch normalization layers can be added right after the dropout layers, and in this case we have given them no additional parameters (although Keras allows for various non-default parameters to be specified, if desired; see Keras documentation for details).

When we run this job file, we get an average test accuracy of 50.6% – nominally a bit better than the previous configuration, but not significantly different (p>.5). It also took slightly longer to run than the previous configuration (about 4.5 minutes). This is perhaps not too surprising, as batch normalization may be more beneficial in networks that are larger and more complex than the one we are using here. So, overall, it may not be worth including batch normalization in our network model. (Although, in this case, it didn't seem to hurt accuracy either.) So, let's forget about batch normalization in the next attempt, and try a different modification to the analysis.

j. For our last tweak, we will try swapping out our optimizer algorithm. Previously, we were using *rmsprop*; now, let's try another popular choice of optimizer, *Adam*.

```
   "compile_options":
   { "loss": "categorical_crossentropy",
    "optimizer": "Adam",
    "optimizer_options":
    { "lr": 0.005,
     "beta_1": 0.9,
     "beta_2": 0.999,
     "epsilon": 0.1,
     "decay": 0
    }
```

As you can see, whereas previously with *rmsprop* we did not bother to specify any additional options beyond the Keras defaults, with *Adam* we have chosen to specify particular non-default values for a few of its options. You can see the Keras documentation on the Adam optimizer for details on what these arguments mean, but for now, suffice to say they are yet another thing that you could try adjusting here and there to see if they make a difference on a particular dataset.

When we run this job file, we get an average test accuracy of 51.3% – a slightly larger improvement over the midsize model with dropout than we got from adding batch normalization, but still not a significant difference (p=.14). For the record, here are summaries of all the variations we ran, which we obtained with the following command:

*python utilities/dt_accs_summarizer.py example_output_folder/*_accs.tsv*

```
delineate — -zsh — 140×32

Summary of: example_output_folder/example_simple_mlp_analysis_largest_accs.tsv
- Nits: 30
- Acc:  Mean =   0.502151,      Stdev =   0.024892,      StdErr =   0.004545
- Loss: Mean =   1.634173,      Stdev =   0.062647,      StdErr =   0.011438

Summary of: example_output_folder/example_simple_mlp_analysis_midsize_accs.tsv
- Nits: 30
- Acc:  Mean =   0.488053,      Stdev =   0.026070,      StdErr =   0.004760
- Loss: Mean =   1.579514,      Stdev =   0.070772,      StdErr =   0.012921

Summary of: example_output_folder/example_simple_mlp_analysis_midsize_dropout_accs.tsv
- Nits: 30
- Acc:  Mean =   0.502151,      Stdev =   0.028133,      StdErr =   0.005136
- Loss: Mean =   1.371694,      Stdev =   0.066528,      StdErr =   0.012146

Summary of: example_output_folder/example_simple_mlp_analysis_midsize_dropout_adam_accs.tsv
- Nits: 30
- Acc:  Mean =   0.513381,      Stdev =   0.030612,      StdErr =   0.005589
- Loss: Mean =   1.643413,      Stdev =   0.054881,      StdErr =   0.010020

Summary of: example_output_folder/example_simple_mlp_analysis_midsize_dropout_batchnorm_accs.tsv
- Nits: 30
- Acc:  Mean =   0.506452,      Stdev =   0.032363,      StdErr =   0.005909
- Loss: Mean =   1.279164,      Stdev =   0.067421,      StdErr =   0.012309

Summary of: example_output_folder/example_simple_mlp_analysis_smallest_accs.tsv
- Nits: 30
- Acc:  Mean =   0.462127,      Stdev =   0.041862,      StdErr =   0.007643
- Loss: Mean =   1.166612,      Stdev =   0.056449,      StdErr =   0.010306
```

k. Looking over our experiments, we have clearly improved performance a bit relative to the very simplest network we tried, but even our best performance value of 51.3% is still far from perfect. Does this mean that about 50% accuracy is the best we can hope for from this dataset? Perhaps… human neuroscience data is intrinsically noisy, and realistically we can probably never hope to get particularly close to 100%. No matter how good our classification is, the fact remains that on some trials, subjects were likely not paying very good attention, or were performing the task poorly in some other way, making the data completely unclassifiable. However, it is very hard to establish what that "noise ceiling" actually is. Perhaps with more changes to the network structure, particularly more drastic changes – adding more layers? swapping some Dense layers out for convolutional ones? – test accuracy might suddenly jump to 60% or higher, for all we know. We will conclude our tutorial here for now, but readers are welcome to continue these explorations on their own.

Overview of Common Network Architecture Choices and Hyperparameters

Here we list some of the most common and important choices one might need to make while designing a neural network architecture for use in dMVPA. There are certainly many more options to choose from than are listed here, and of course, when we consider the space of all possible *combinations* of these options, the number of possible architectures is effectively infinite. As discussed above, we typically recommend starting with a relatively small/simple network architecture and/or performing a traditional non-deep MVPA to establish a performance baseline, and then experimenting with different hyperparameters and larger/more complex network structures from there. For a full rundown of the many available hyperparameters, please consult the DeLINEATE toolbox documentation (for options specific to this toolbox), the online Keras documentation, and any of the many online guides on getting started with deep learning.

**Layer type**	**Dense**: The classic artificial neural network layer, as used (for example) in a multi-layer perceptron network configuration. For a classification task, the final layer in the network will generally be a Dense layer. Many other network architectures will feature one or more additional Dense layers as well. Although Dense layers might be thought of as a relatively basic layer type, keep in mind that they tend to increase the number of trainable parameters fairly drastically, especially when placed directly after a large input. (For example, a Dense layer with 12 units that receives a 1000-length vector as input will have 12,000 weights to train.) Relevant other hyperparameters for Dense layers include regularization, activation function, and number of units.
	**Convolutional**: There are several different subtypes of convolutional layer in Keras according to the dimensionality of the input (**Conv1D**, **Conv2D**, **Conv3D**). Typically, most users will probably stick to those basic types, although other, more arcane convolutional layer types also exist in Keras. It is not uncommon to have one or more convolutional layers in a network, followed by one or more Dense layers before the output; if using an architecture like this with 2-D or 3-D data, one must remember to flatten the data with a Flatten layer (see Keras documentation and DeLINEATE toolbox sample job files for usage) between the convolutional and Dense layers. Relevant hyperparameters for convolutional layers include the number of convolutional filters to train per layer (each of which will result in creating a copy of the transformed input data as output; keep this in mind if following a convolutional layer with a Dense layer, which can result in large parameter counts); the kernel size (i.e., the size of the digital filter that is trained); the stride (how many units of input data to skip in each dimension when moving the filter over the data); and activation function.
	**Recurrent**: There are also several different subtypes of recurrent layer in Keras, with perhaps the most common choices being **LSTM** (long short-term memory) and **GRU** (gated recurrent unit); see the Keras documentation for the full list. Recurrent layers have an internal “memory” of sorts and thus are typically useful with timeseries data where interpretation of one timepoint is likely to be informed by previous timepoints in the signal. Relevant hyperparameters for recurrent layers include *return_sequences* (whether to return an output value for each element of the input sequence, or only the final timepoint); the number of units (similar to convolutional layers, keep in mind that each unit will produce a full copy of the input data, assuming that *return_sequences* is True); and activation function.
**Regularization**	**Weight regularizers**: Keras has several different options for applying regularization to different aspects of a layer, but the one we use most frequently is the *kernel_regularizer*, which applies regularization to the weights of the units. You can choose from L1 regularization, L2 regularization, or a combination of both. It may take some experimenting to find an optimal setting, but typically these should be small values, on the order of around .01 or smaller.
	**Batch normalization**: This regularization technique essentially standardizes the mean and standard deviation of the inputs to a layer during each batch of training. Theoretically, this should potentially speed up training, possibly increase accuracy, and make the training process less sensitive to the initial randomized values of the network’s weights. In practice, it may or may not help to add batch normalization between your layers, depending on the dataset and network architecture. Keras has several hyperparameters that can be specified for a batch normalization layer, although when we use it, we typically omit those values and simply let it use the default settings.
	**Dropout**: This method randomly drops a certain percentage of units during training, which injects some unpredictability into the training process and has a net regularizing effect. The main hyperparameter to worry about is the *rate* value, which should be between zero and one, representing what proportion of units to drop. Reasonable values would probably range between about 0.1 and 0.3, generally speaking.

	**Early stopping**: This method also helps to limit overfitting by cutting off training when performance on a held-out validation dataset stops improving for a specific number of training epochs. (By avoiding unnecessary overtraining, it also just helps analyses run faster.) Early stopping is technically optional in Keras, but for dMVPA it is sufficiently universally useful that DeLINEATE assumes by default that it will be used. The main hyperparameter to worry about is *patience*, which is how many epochs of non-improved validation to run before stopping. Reasonable values might be anywhere from tens to hundreds of epochs, depending on how quickly your network training tends to converge. This value is relatively easy to determine with trial-and-error; simply set it very high, start the analysis, observe about how many epochs it takes for validation performance to asymptote, force-quit the analysis, and repeat. After observing a handful of times, try to set the patience to a value that tends to cut off training around the same number of epochs as you observed the asymptote.
**Activation function**	**ReLU (and variations)**: ReLU stands for rectified linear unit, and some form of ReLU is probably the most common and safest activation function to use for most purposes. Other variants include **PReLU** and **LeakyReLU**; we mostly use LeakyReLU. You may not need to specify any particular hyperparameters if using a plain ReLU. If using a LeakyReLU, you may want to specify *alpha*, which controls the slope of the function for input values < 0; we typically use *alpha* values around 0.1.
	**Others**: As we have noted elsewhere, **softmax** is the activation function you will probably use for your output layer, but not other layers. Other possible choices you could try out might include **tanh** and **sigmoid**; note that these last two are available as values that can be passed to the *activation* hyperparameter of a primary layer type, but they cannot be specified as standalone layers the way that **ReLU** and its variants can.
**Other computation layers**	**Max pooling**: Sometimes, you may want to downsample either your input data or an intermediate output of a layer of your neural network. One way to do this is with max pooling, which replaces a set of values with their single maximum value. There are variations (**MaxPooling1D**, **MaxPooling2D**, **MaxPooling3D**) for different dimensionalities of input data. The main hyperparameters to worry about are probably *pool_size* (essentially, the size of the moving window over which the max function is run) and *strides* (how far to move the window).
	**Average pooling**: For an even more conventional form of downsampling, there is average pooling. This has the same kind of variations (**AveragePooling1D**, **AveragePooling2D**, **AveragePooling3D**) as max pooling, but the output is the average value of the inputs inside a moving window, rather than the max. Again, the main hyperparameters are *pool_size* and *strides*, which work the same way as in max pooling.
**Number/order of layers**	Obviously a critical choice, but one that is hard to recommend rules-of-thumb for, due to the wide variety of datasets and deep learning approaches that exist in the world. Historically, the "deep" in "deep learning" derived from networks with many layers, but these days the name is taken less literally; one can often get quite decent "deep learning" results with only a handful of layers. For a typical dMVPA classification analysis, these layers could all be Dense (i.e., a multi-layer perceptron); they could all be convolutional (except for a final Dense layer used for output values); they could all be recurrent (again, except for a final Dense layer), or they could be a mix: A few convolutional layers followed by a few Dense, or a few recurrent layers followed by a few Dense, would be the most straightforward, but much more exotic combinations are possible if you have enough data and enough time to experiment with.
**Number of units/filters per layer**	Another critical choice that clearly affects your network's ability to learn. Larger layers theoretically have more learning potential, but also with increasing complexity can come (sometimes) increased propensity to overfit, as well as increased computation times. Again, it is hard to offer concrete advice that is universal across datasets, other than perhaps to start with smaller numbers of units/filters and gradually increase until benefits tail off and signs of overfitting start to appear. Sometimes you might want to have more units in earlier layers of your network and fewer in later layers, sometimes the reverse, and sometimes you might want to have about the same layer size throughout. Experimentation is again your friend, although it certainly helps if you can start from a configuration that has been known to work well with similarly structured datasets, and branch out from there.
**Optimizer**	Optimizers are the functions that actually control how layer weights are updated; common choices might include **SGD**, **RMSprop**, and **Adam**, although there are several more supported by Keras that you could play around with. All have a *learning_rate* hyperparameter that controls the initial rate of how aggressively synaptic weights are updated with each pass of training; the learning rate typically defaults to .001 in most cases, but you could experiment with adjusting it, perhaps up to about a degree of magnitude or two in either direction. Different optimizers have additional hyperparameters that you may wish to tweak as well; please consult the Keras documentation for details on those.

Note: All subsequent rows of this table are DeLINEATE toolbox settings only, not hyperparameters found in Keras	
**Number of iterations**	This value (called *nits* in our job files) controls how many iterations of cross-validation to run, where each iteration splits a full dataset into different random training/validation/test subsets, retrains a new model on the training dataset, and tests it on the test dataset. Reasonable values of *nits* depend somewhat on how your dataset is configured (e.g., is it all from one subject, or from many subjects?) and the cross-validation type used (see below), but generally you want enough iterations that your mean test accuracy appears stable (i.e., has a sufficiently small standard error). We typically run from 10 to 50 iterations in our own datasets.
**Cross-validation type**	**single**: Also called a “universal” analysis in some of our documentation, this cross-validation type treats all input data as a single pool. This might mean it’s from a single subject, or it could mean that you have a number of unique subjects but don’t mind lumping them all in together. It would also work for non-human, non-animal data, such as a set of photographs or other common deep learning data types.
	**loop_over_sa**: This cross-validation type performs the specified analysis once for every unique value in a specified “sample attribute” of the dataset. Most frequently, this would be used to do a separate analysis for each subject/participant in a dataset.
	**Future**: This is not an actual cross-validation type, but a note to check the DeLINEATE website for updates to the toolbox, as additional types of cross-validation are planned for future versions.
**Train/val/test ratio**	This refers to what proportion of the input dataset should be used for training, validation, and test subsets, respectively. This could vary widely depending on intended application, but as a general rule of thumb, previous classical MVPA guidelines have often recommended train/test splits on the order of perhaps 70/30 to 90/10. Extrapolating to add in a validation set, we most frequently use splits on the order of [60,20,20] or [70,15,15], although this can be adjusted; if you have very limited data and need most of it for training, for example, you could go with something like [80,15,5]. This would make the test dataset very small and hence test accuracy quite variable, but you could offset that variability by increasing the number of iterations until the mean test accuracy across iterations appears stable.
**Scaling**	DeLINEATE offers the convenience utility to scale your input data for you, as most traditional MVPA methods are not particularly sensitive to the scale/range of the input data, but deep learning can sometimes be significantly more sensitive to it. (Again, standard caveat: The magnitude of such sensitivity can depend quite a bit on the exact configuration of your network hyperparameters.) These methods should all be safe from double-dipping concerns by defining scales only on the training dataset without peeking at the validation or test datasets. Possible options include **percentile** (rescale all data according to the Nth percentile of the absolute value of all training data points), **standardize** (Z-score all data based on the mean and standard deviation of the training data points), **map_range** (map all values into the range 0-1, or another user-specified range), and **mean_center** (simply remove the mean but do not divide by any factor). Please see the DeLINEATE documentation for exact usage information.

Supplementary Methods: Benchmark Data Generation

Please note that for any readers who wish to recreate the benchmark data discussed in the text, you can find it by first downloading the DeLINEATE toolbox, unzipping it, and looking in the *sample_data* folder and then the *benchmark_data* sub-folder. In *benchmark_data* you will find the file *benchmark_data_generator.m*, with some default settings that are slightly different than what were used in the paper. To recreate the benchmark dataset from the paper, two small changes should be made: change the value of the *n_features* variable at the top of the script to *[200 400 800 1600 3200 6400 12800 25600]* (which will generate data for the full range of feature sizes included in the paper), and set the value of the *big_file_format* variable to *true* (which will ensure that Matlab saves the data out in a format that can handle larger file sizes). If you then run the script in Matlab, it should recreate the benchmark dataset. (If you do not have access to Matlab, a commercial application, the script may also work in the open-source Octave; we have not tested it in Octave, but none of the code is particularly exotic, and thus it is likely to work in Octave with little modification.)

As discussed in the text, we simulated datasets with three conditions (classes). The number of features (e.g., simulated voxels) in the datasets ranged from 200 to 25,600, in the doubling progression listed above. The number of examples (aka trials) per condition ranged from 100 to 10,000, drawn from the set: [100 400 900 1600 2500 3600 4900 6400 8100 10000]. The script also contains two "noise coefficient" variables that control the amount of noise added to the data; *noise_coef1* was set to 5000 and *noise_coef2* to .8. A dataset was generated for every combination of feature sizes and number of examples.

The actual dataset generation proceeded as follows; suppose, for this example, that we are generating the dataset with 800 features and 3600 trials per condition. For each condition, first a "base" signal is created of 800 random values in the range [-1, 1] (uniform distribution). This base signal can be considered the "ground truth" for a hypothetical voxel pattern corresponding to that condition (i.e., the ideal voxel pattern we would expect by averaging an infinite number of trials). Then, we create 60 "Level 1" variations of the base signal; each Level 1 variant is obtained by adding another random signal the same size as the base signal (but using a Gaussian distribution), multiplied by *noise_coef1*, to the base signal. Then, we repeat this process to create 60 "Level 2" variants of each Level 1 variant. E.g., in pseudocode:

```
for i = 1 to 60
  level_1_variant(i) = base_signal + generate_random_vector(800) * noise_coef1
  for j = 1 to 60
    level_2_variant(i,j) = level_1_variant(i) + generate_random_vector(800) * noise_coef1
```

Although this process is not meant to be incredibly biologically plausible, the rough central idea is is that in real-life data, there might be a handful of common variations on the base signal – for example, different gender/race/age combinations for a main category of "face," if this were data from a visual fMRI study. Those are the Level 1 variants. The Level 2 variants simulate random fluctuations in signal from trial to trial within each Level 1 variant. For simplicity, we "branch" the same number of variants at each level, and also add the same proportion of Gaussian noise at each level, although there is no particular reason this would need to be the case, aside from trying to minimize the number of variables in play in the script.

The Level 2 variants will ultimately form the basis for the benchmark trials, but one more step is required to make them sufficiently noisy to provide a challenge for a competent classifier. Once all of the Level 2 variants for all conditions (classes) have been generated, each of these proto-trials is blended with a mixture of signals from a randomly selected proto-trial from each of the other two conditions, according to the proportion specified by *noise_coef2*. With *noise_coef2* set to .8, this means that a trial of Condition A would consist of 20% signal from a proto-trial from Condition A, 40% from a randomly selected proto-trial from Condition B, and 40% from a randomly selected proto-trial from Condition C.

Appendix: Contents of sample JSON job file *example_simple_mlp_analysis_smallest.json*


```
[
  { "model":
    { "layers":
      [
        { "input_shape": [ 18, 273 ],
          "layer_type": "Reshape",
          "target_shape": [ 4914 ]
        },
        { "layer_type": "Dense",
          "units": 5,
          "kernel_regularizer": "keras.regularizers.l2(0.01)"
        },
        { "layer_type": "LeakyReLU",
          "alpha": 0.1
        },
        { "layer_type": "Dense",
          "units": 3,
          "kernel_regularizer": "keras.regularizers.l2(0.01)"
        },
        { "layer_type": "LeakyReLU",
          "alpha": 0.1
        },
        { "layer_type": "Dense",
          "activation": "softmax",
          "units": 3
        }
      ],
      "compile_options":
      { "loss": "categorical_crossentropy",
        "optimizer": "rmsprop"
      }
    },
    "data":
    { "loader_params":
      [ "sample_data/sample_fso_dataset_vsl_study.mat",
        "erp_data",
        "categories"
      ],
      "loader": "sample_generic_matfile_loader"
    },
    "analysis":
    { "nits": 30,
      "xval_type": "single",
      "train_val_test": [ 70, 15, 15 ],
      "scaling": 90,
      "classify_over": "class",
      "backend_options":
      { "patience": 50,
        "epochs": 10000,
        "batch_size": 500,
        "verbose": 1
      }
    },
    "output":
    { "output_filename_stem": "example_simple_mlp_analysis_smallest",
      "output_location": "example_output_folder",
      "output_file_types": "all"
    }
  }
]
```
